# Neurocomputational mechanisms underpinning aberrant social learning in young adults with low self-esteem

**DOI:** 10.1038/s41398-020-0702-4

**Published:** 2020-03-17

**Authors:** Geert-Jan Will, Michael Moutoussis, Palee M. Womack, Edward T. Bullmore, Ian M. Goodyer, Peter Fonagy, Peter B. Jones, NSPN Consortium, Robb B. Rutledge, Raymond J. Dolan

**Affiliations:** 1 Max Planck University College London Centre for Computational Psychiatry and Ageing Research, London, UK; 2 Wellcome Centre for Human Neuroimaging, University College London, London, UK; 3 Institute of Psychology, Leiden University, Leiden, The Netherlands; 4 Department of Psychiatry, University of Cambridge, Cambridge, UK; 5 Cambridgeshire and Peterborough National Health Service Foundation Trust, Cambridge, UK; 6 Research Department of Clinical, Educational and Health Psychology, University College London, London, UK

**Keywords:** Neuroscience, Psychology

## Abstract

Low self-esteem is a risk factor for a range of psychiatric disorders. From a cognitive perspective a negative self-image can be maintained through aberrant learning about self-worth derived from social feedback. We previously showed that neural teaching signals that represent the difference between expected and actual social feedback (i.e., social prediction errors) drive fluctuations in self-worth. Here, we used model-based functional magnetic resonance imaging (fMRI) to characterize learning from social prediction errors in 61 participants drawn from a population-based sample (*n* = 2402) who were recruited on the basis of being in the bottom or top 10% of self-esteem scores. Participants performed a social evaluation task during fMRI scanning, which entailed predicting whether other people liked them as well as the repeated provision of reported feelings of self-worth. Computational modeling results showed that low self-esteem participants had persistent expectations that others would dislike them, and a reduced propensity to update these expectations in response to social prediction errors. Low self-esteem subjects also displayed an enhanced volatility in reported feelings of self-worth, and this was linked to an increased tendency for social prediction errors to determine momentary self-worth. Canonical correlation analysis revealed that individual differences in self-esteem related to several interconnected psychiatric symptoms organized around a single dimension of interpersonal vulnerability. Such interpersonal vulnerability was associated with an attenuated social value signal in ventromedial prefrontal cortex when making predictions about being liked, and enhanced dorsal prefrontal cortex activity upon receipt of social feedback. We suggest these computational signatures of low self-esteem and their associated neural underpinnings might represent vulnerability for development of psychiatric disorder.

## Introduction

Low self-esteem is a core symptom of a range of common mental health problems^1,2^. People with low global self-esteem, an overall negative evaluation of self-worth, exhibit cognitive biases that are thought to contribute to the maintenance of a negative self-image. Those with low self-esteem have expectations that others will view them in a negative light^3,4^ and their feelings of self-worth are more responsive to social feedback^5^. Persistent negative self-views and instability in feelings of self-worth are linked to onset and maintenance of psychiatric disorders, including depression^6,7^, anxiety^2^^,8^ and psychosis^9,10^. Here, we use computational modeling and functional magnetic resonance imaging (fMRI) to ask how low global self-esteem impacts on learning about the self during social evaluation.

How we appraise ourselves arises, in part, out of beliefs we hold regarding how others view us. Appraisals from close others are important developmental building blocks in constructing a sense of self-worth when negotiating childhood and adolescence^11–13^. When children repeatedly receive feedback that they are not worthy, they are prone to develop a chronic negative view of the self (a negative “direct self-appraisal”) and a persistent belief that others will not approve of them (a negative “reflected self-appraisal”)^3,11,13^. We recently developed a computational model of self-esteem where we showed human subjects exploit neural teaching signals, representing a difference between expected and actual social feedback (i.e., social approval prediction errors or SPEs), to learn about their social standing as expressed in reported self-worth^14^. People use SPEs to update expectations about whether others like them (i.e., “reflected” self-appraisals) and to simultaneously update subjective feelings as to how much they value the self (i.e., “direct” self-appraisals). Here, we extend this work by examining whether in subjects with low self-esteem persistence of negative expectations about social evaluation, and an increased reactivity in reported feelings of self-worth in response to social feedback, are explained by aberrant weighting of SPEs.

In a sample of subjects with average to high self-esteem, SPEs correlated with activity in ventral striatum and subgenual anterior cingulate cortex (VS/sgACC), while updates in self-worth were reflected in ventromedial prefrontal cortex (vmPFC) activity^14^. Ventral striatum has been shown to encode prediction errors used during social and non-social learning^15–18^, while sgACC is suggested to encode a domain-specific social learning signal^18–20^. Activity in vmPFC has been consistently shown to represent subjective value, both at decision time and at decision outcome^21–23^. The vmPFC is also implicated in making evaluations about the self and other people^23^. An anterior subportion of vmPFC (BA 11) is reported to support self- and other-directed cognition that attenuates the impact of negative social feedback on self-worth^24–26^, rendering it a candidate region for explaining individual differences in learning from social feedback.

The goal of the current study was to characterize the neurocomputational basis of learning biases that are thought to contribute to a development of mental health problems in those with low self-esteem. We employed a targeted recruitment approach involving selecting participants from a large community sample (*n* = 2402)^27^ scoring within the bottom or top 10% of global self-esteem scores, but who had no concurrent diagnosis of psychiatric disorder. This focus on the extremes of a reported self-esteem distribution, and its naturally co-morbid symptomatology, enables sampling a greater individual variation than can be obtained by sampling randomly from the population (Fig. S1). This afforded an investigation of learning from social feedback in low self-esteem individuals with substantial subclinical mental health problems, but who were free of common confounds associated with patient samples (e.g., contamination by interventions, medication, or stigma). Rather than comparing these subjects to an average self-esteem group (who have average levels of symptoms), we contrasted them with a group of high self-esteem individuals based on the well-established notion that high self-esteem individuals have lower levels of psychiatric symptoms, including anxiety and depression^2^, higher levels of well-being^28^ and are more resilient to social stressors^5^ than people with average self-esteem. This was also the case in the present study where high self-esteem participants ranked among the highest in well-being and lowest in depression within the large community sample (*n* = 2402) from which they were selected (Fig. S2).

We predicted low self-esteem would be associated with persistent negative expectations about future social feedback, and an increased responsivity to social feedback as expressed in reported feelings of self-worth^5^. Based on our prior work on self-esteem, we hypothesized that such individual differences would be attributable to an aberrant weighting of social approval prediction errors^14^. This prior work led us also to predict the expression of neural signatures of social approval prediction errors in VS/sgACC and updates of self-worth in vmPFC. In addition to examining categorical differences between high and low self-esteem participants, we also employed a dimensional approach. Previously, we showed that individual differences, in both computational and neural processes, underpinning learning about self-worth could be captured by a dimensional marker of “interpersonal vulnerability”^14^. Participants scoring high on this dimension showed specific computational features (e.g., increased dependence on SPEs for self-worth), elevated interpersonal and psychiatric problems and enhanced prediction error processing in anterior insula (but not VS/sgACC). Here, we assessed whether we could replicate this dimension across the entire self-esteem spectrum (now including subjects with very low self-esteem), and whether very low self-esteem is associated with distinct neural signatures during learning about self-worth.

## Materials and methods

### Participants

We recruited human subjects from a large population-representative sample of young people in London and Cambridge areas (NSPN 2400 Cohort; *n* = 2402^27^) who reported on their mental health across 1–3 measurements spanning 4.5 years. For the current study, we selected participants based on global self-esteem scores on the Rosenberg self-esteem scale (RSES^29^, which measures a person’s evaluation of their overall self-worth). Participants were matched for age and gender, but not for subclinical measures of distress that co-vary with low self-esteem, such as symptoms of depressed mood and anxiety to maximize ecological validity^28^ (Table 1).

**Table 1:** Participant characteristics.

	Group		
Characteristic	Low self-esteem (*n* = 30)	High self-esteem (*n* = 31)	Statistical test and *p*-value*
Global self-esteem score on day of scanning^29^, Median (IQR)	15.0 (0.85)	28.0 (0.50)	*z* (59) = −6.17, *p* < 1 × 10^−9^
Female, No (%)	18 (60%)	16 (52%)	*χ*^2^ (1) = 0.435, *p* = 0.510
Age, Median (IQR)	21.3 (1.94)	20.9 (2.34)	*t* (59) = 0.77, *p* = 0.168
Ethnicity, No (%)	White: 17 (57%) Black: 0 (0%) Asian: 10 (33%) Mixed: 2 (7%) Other: 1 (3%)	White: 20 (65%) Black: 2 (7%) Asian: 5 (16%) Mixed: 3 (10%) Other: 1 (3%)	*χ*^2^ (1) = 0.394, *p* = 0.530
Rejection sensitivity score^41^, Median (IQR)	11.22 (4.67)	6.67 (2.72)	*z* (59) = −4.52, *p* < 1 × 10^−4^
Fear of negative evaluation score^40^, Median (IQR)	3.33 (1.46)	1.83 (1.15)	*z (*55) = −5.10, *p* < 1 × 10^−5^
State anxiety score^42^, Median (IQR)	1.70 (0.65)	1.20 (0.45)	*z* (59) = −4.43, *p* < 1 × 10^−4^
Trait anxiety score^42^, Median (IQR)	2.65 (0.83)	1.48 (0.38)	*t* (59) = −5.69, *p* < 1 × 10^−7^
Social anxiety score^43^, Median (IQR)	0.94 (0.82)	0.55 (0.64)	*t* (55) = −2.43, *p* = 0.015
Depressed mood score^44^, Median (IQR)	21.00 (21.50)	6.00 (9.00)	*t* (59) = −4.86, *p* < 1 × 10^−5^

*IQR* interquartile range.

*p-values obtained using Mann–Whitney *U* tests (when data were not distributed normally), independent samples *t*-tests (when data were distributed normally), and Chi-square tests for gender (male vs. female) and ethnicity (white vs. non-white).

Mean RSES score of the large sample was 19.7 (on a scale of 0–30; SD = 5.62; Fig. S1). We invited 184 participants with average RSES scores within the bottom decile (0–12) and top decile (27–30) of the large sample for further study and scanned 53 participants (29 low self-esteem; 24 high self-esteem). To reach our target sample size of 30 subjects in each group, we invited a further 51 subjects whose recent RSES score was within the bottom or top decile of RSES scores and scanned an additional 10 of these. Sample size was chosen to exceed the number of participants in prior fMRI studies examining inter-individual differences in self-esteem (10 studies; median *n* = 26; range = 17–48)^4,30–38^. Those not incorporated in the MRI study did not differ from MRI participants either in terms of average RSES score, recent RSES score, age, or gender (all *p*s > 0.17).

Additional inclusion criteria included: absence of current psychiatric or neurological disorder, an address in London, absence of color blindness, and no contraindications that prohibited MRI scanning (e.g. metal implants). While a current diagnosis of psychiatric disorder was an exclusion criterion, subjects were allowed to participate if they had a history of psychiatric illness and had been in remission for at least 3 years. Five low self-esteem participants reported having recovered from a mental health problem at least 3 years prior to the MRI scans (depression and anxiety: *n* = 2, depression: *n* = 2, anorexia nervosa: *n* = 1). Two participants were excluded because they did not finish the experiment due to equipment failure.

The final sample comprised 30 low self-esteem participants (mean age = 21, SD = 1.9; 18 females) and 31 high self-esteem participants (mean age = 21, SD = 2.3; 16 females) who received £8 per hour of participation, earnings based on an additional task (Dictator Game; see Fig. S7), and compensation for travel expenses. Self-esteem data used for recruitment was on average collected 27.6 months prior to acquisition of the MRI scans (SD = 9.2; range = 12–52 months). There was no relationship between months elapsed since last self-esteem assessment and global self-esteem score at the time of MRI scanning (*p* = 0.357) or changes in self-esteem since last self-esteem assessment (*p* = 0.243). The study was approved by the London—Westminster NHS Research Ethics Committee (15/LO/1361). All participants gave written informed consent.

### Procedure

After initial screening over the phone, participants were asked to create an online character profile about their personality as well as their likes and dislikes (Table S1). Participants were told their character profile would be uploaded to an online database where other people, between the ages of 18 and 25, could see their profile. These raters would then evaluate the profile and decide whether they would be interested in becoming friends with the participants if they met them in real life. Researchers involved in data collection knew that recruitment was based on participants having either high or low global self-esteem, but they were blinded to individual participants’ self-esteem level during data collection.

Participants attended the lab at least 5 days after creating their profile (mean = 19.6, SD = 21.4) so as to allow sufficient time to pass needed for collecting enough evaluations for the experiment. On the day of testing, they received task instructions and practiced a few trials of the social evaluation task they would perform in the scanner (see below for details). Before practicing the task, they were shown an online forum where raters purportedly evaluated their profile. In reality, the task feedback they received was generated by an algorithm independent to their profiles. After scanning, they performed a control experiment (Supplementary Results) as well as completed a funneling suspicion probe to assess whether participants believed the feedback was derived from authentic appraisals of other people (see Supplementary Materials). Only one high self-esteem participant and only one low self-esteem participant raised doubts about authenticity of social feedback. Both participants exhibited higher self-worth after approval and lower self-worth after disapproval (both *B*s > 0.03, both *p*s < 0.023). All behavioral and neuroimaging findings remained significant after excluding these two participants from our analyses, except for a correlation between updating-related activity in dPFC and interpersonal vulnerability. However, a partial correlation analysis showed that the correlation between dPFC activity and interpersonal vulnerability remained significant (*ρ*(58) = 0.26, *p* = 0.049 after controlling for doubts about the cover story, suggesting that this result was not confounded by the expression of doubt about the cover story. After being debriefed about the cover story, the participants were given a break after which they filled out questionnaires assessing symptoms associated with low self-esteem.

### Social evaluation task

Participants performed a task involving receipt of approval and disapproval feedback from 184 raters who ostensibly evaluated participants’ online character profile (see Supplementary Methods)^14^. Raters were ordered into four groups based on their general propensity to positively or negatively evaluate participants in the study. Feedback was pre-programmed such that the probability of receiving approval feedback depended on rater’s group membership, with specific rater approval feedback generated in 87%, 67%, 33%, and 13% of trials. Participants were not instructed about these exact probabilities, but learned the rank ordering of the rater groups before performing the task. On each trial, participants were presented with the name of a rater and a color cue that indicated the rater’s group membership (Fig. S3). Participants could then indicate whether they expected to be liked by the rater before receipt of either approval (“a thumbs up symbol”) or disapproval feedback (“a thumbs down symbol”). After every 2 to 3 choice trials, participants reported their self-worth using a visual analog scale from 0 to 1 (75 ratings).

### Psychiatric symptom measures

To characterize behavioral variability across both computational self-esteem parameters and psychiatric symptoms, we assessed self-reported symptoms of global self-esteem, interpersonal sensitivity, anxiety, and depressed mood on the day of scanning. Global self-esteem was assessed using the RSES^29^. Interpersonal sensitivity measures included the Brief Fear of Negative Evaluation scale^39,40^ and the Rejection Sensitivity Questionnaire^41^. Anxiety measures included the State and Trait Anxiety Inventory^42^, and the Liebowitz Social Anxiety Scale^43^, and depressed mood was assessed with the Mood and Feelings Questionnaire^44^.

### fMRI data acquisition and analysis

MRI scans were acquired using a 3T Siemens Trio MRI scanner (Siemens Healthcare) and a 32-channel head coil. We used a blood oxygenation level-dependent (BOLD) sensitive T2*-weighted single shot echo-planar imaging sequence optimized to minimize signal dropout in striatum and ventral frontal cortex^45^. We used a pulse-oximeter and breathing belt to collect physiological data to correct for physiological noise in fMRI analyses. The task was presented in MATLAB (MathWorks, Inc.) using Cogent 2000 (Wellcome Centre for Human Neuroimaging) and projected onto a screen in the magnet bore. Participants could see this screen through a mirror attached to the head coil. They could respond to the stimuli by pressing buttons on a fiber optic response box using their right index and middle finger. Head motion during scanning was restricted using foam inserts.

MRI data were preprocessed and analyzed using SPM12 (Wellcome Centre for Human Neuroimaging, University College London). Functional MR images were slice-time corrected, corrected for field-strength inhomogeneities using field maps, unwarped and realigned, co-registered to subject-specific structural images (magnetic transfer images maps acquired using quantitative multiparameter maps; see Supplementary methods), normalized to MNI space (using the DARTEL toolbox^46^) and smoothed using a 8-mm, full-width at half-maximum isotropic Gaussian kernel.

We used our computational model to examine BOLD responses that scaled parametrically with three variables of interest: (1) expected social value (ESV) at time of choice, (2) social approval prediction errors (SPEs) upon receipt of feedback, and (3) self-worth updates upon receipt of feedback. Following a common procedure in computational fMRI studies of individual differences^35,36^, model-based parametric modulators were generated by applying mean group parameters to individual participants’ sequences of stimuli. We utilized the same two generalized linear models (GLMs) that we deployed in our previous study on self-esteem in the general population^14^. To examine neural representations of ESV and SPEs, we constructed a GLM with regressors indicating cue onset, delay period, social feedback onset, self-worth probe question onset and button press onset for provision of a self-worth rating. The cue onset regressor was parametrically modulated by ESV, the feedback onset regressor was parametrically modulated by SPEs, and the self-worth question onset regressor was parametrically modulated by z-scored self-worth rating. All events were modeled as stick functions with 0 s duration.

To examine neural signatures of self-worth updates at time of feedback, we constructed a similar GLM. However, in this GLM both cue and feedback regressors were parametrically modulated by self-worth updates inferred using our computational model (instead of ESV and SPE). Both models also contained six regressors to correct for motion-induced noise (based on the realignment parameters) and 18 cardiac and respiratory regressors to correct for physiological noise. Subject-specific contrast images were submitted to group level random-effects analyses.

### Statistical analysis

For analyses of behavior, we used non-parametric tests that do not assume data are normally distributed, including Mann–Whitney *U* test and Spearman correlations. Significance was set at *p* < 0.05 (two-tailed). Neuroimaging results were corrected for multiple comparisons with Family-wise Error (FWE) cluster-correction at *p* < 0.05 (cluster-forming threshold of *p* < 0.001). For all behavioral and neuroimaging analyses, we first tested for categorical differences between the high and low self-esteem groups using Mann–Whitney *U* tests (behavioral data) or independent samples *t*-tests (neuroimaging data). Subsequently, motivated by our prior work, and that of others^47–49^, we employed a dimensional approach to test for continuous associations between low self-esteem and brain and behavior. Here, we first characterized the dimensionality of self-reported psychiatric symptoms and computational self-esteem parameters using a canonical correlation analysis (CCA) across the entire sample (*n* = 61). We replicated findings from our prior work that showed symptoms and computational parameters loaded on a single canonical dimension of “interpersonal vulnerability”, where those scoring higher on this dimension report higher symptoms levels and exhibit a computational phenotype associated with vulnerability.

Next, we performed whole-brain analyses testing for an interaction between the resulting mode of co-variation (i.e., interpersonal vulnerability) and brain activity associated with expected social value and self-worth updates. Finally, we correlated vulnerability scores against activity in brain regions functionally involved in representing ESV at choice or self-worth updates upon receipt of feedback identified in whole-brain analyses across the entire sample (see refs. ^50–52^ for a similar approach). For this analysis we used the Marsbar toolbox^53^ to extract activity from two regions of interest (ROIs) where activity positively scaled with self-worth updates (vmPFC; peak coordinates: −3,47,−11 and dorsal prefrontal cortex; −23,29,51; a whole-brain analysis of ESV did not result in group-wise clusters of activation). When testing for replications of our prior neuroimaging results, we used independently defined functional ROIs (6 mm spheres) surrounding peak voxels (ventral striatum/subgenual anterior cingulate cortex: 5,20,−8 and anterior insula: −44,11,9) derived from a prior study using a similar paradigm in an independent sample^14^.

## Results

### Behavioral results

#### Expectations about being liked

**Fig. 1:**
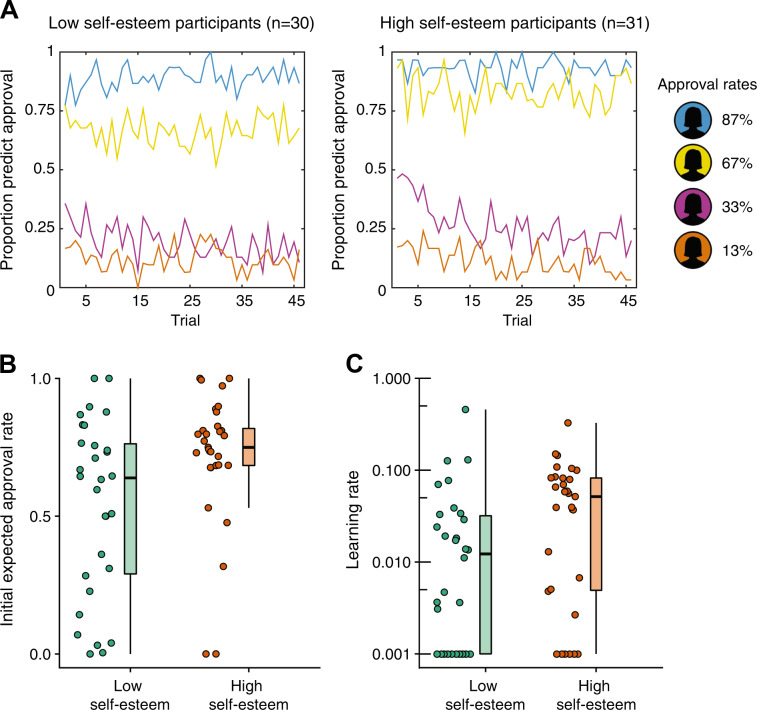
Persistent negative expectations about being liked in low self-esteem participants. **a** Participants with low self-esteem (*n* = 30) predicted being liked less often (main effect of global self-esteem, *p* = 0.017) and did not change their predictions as the experiment progressed (effect of trial not significant, *p* = 0.185). This contrasted with high self-esteem subjects (*n* = 31) who changed their predictions appropriately in response to feedback (significant effect of trial, *p* < 1 × 10^−4^). **b** Low self-esteem participants (*n* = 30) had lower initial expectations about whether other people would like them compared to high self-esteem participants (*n* = 31; Mann–Whitney *U* test, *z* = −2.29, *p* = 0.022); **c** Subjects with low self-esteem had lower learning rates for social approval prediction errors (Mann–Whitney *U* test, *z* = −2.30, *p* = 0.021) indicating they were slower to update their expectations about how much others value them in response to social approval prediction errors. A logarithmic scale is used. Middle line of boxplots represents median and the lower and upper hinges of the boxes correspond to the first and third quartiles. The upper and lower whiskers extend from the hinge to the largest or smallest value respectively no further than 1.5 × interquartile range from the hinge. Figures created using code for Raincloud plots^66^.

We first tested whether participants with low and high self-esteem differed in the predictions they made about being liked. In a generalized linear mixed logistic regression model we assessed the influence of rater group (4 levels: 87%, 67%, 33%, and 13% approval), global self-esteem (2 levels: high and low) and trial number on participants’ predictions. This analysis showed that rater groups influenced predictions of being liked (main effect rater group: *B* = 1.93, SE = 0.03, *χ*^2^(3) = 5883.47, *p* < 1 × 10^−15^) and that participants adapted their responses to feedback as the experiment progressed (main effect trial number: *B* = −0.11, SE = 0.03, *χ*^2^(1) = 14.97, *p* < 1 × 10^−4^;Fig. 1a).

Low self-esteem participants predicted they would be liked less often (47%) than was the case for high self-esteem participants (53%, *B* = 0.22, SE = 0.09, *χ*^2^(1) = 5.47, *p* = 0.017) despite receiving equivalent feedback (50% approval collapsed across rater groups). A significant interaction between global self-esteem and rater group (*B* = −0.08, SE = 0.03, *χ*^2^(3) = 41.78, *p* < 1 × 10^−8^) indicated this difference was greater for certain rater groups. Evaluating the effect of global self-esteem for the 4 rater groups separately showed that low self-esteem participants predicted they would be liked less (66%) than high self-esteem participants (83%) by raters from the mildly positive 67% group (*B* = 0.74, SE = 0.25, *χ*^2^(1) = 8.39, *p* = 0.003), but not from the other groups (all *p*s > .175).

A significant interaction between trial and global self-esteem (*B* = −0.08, SE = 0.03, *χ*^2^(1) = 6.11, *p* = 0.008) showed that low and high self-esteem participants differed in how they learned within the task. Follow-up comparisons showed that low self-esteem participants failed to change their predictions about being liked as the experiment progressed (*B* = −0.04, SE = 0.03, *χ*^2^(1) = 1.76, *p* = 0.185), while a significant effect of trial number was evident in high self-esteem participants (*B* = −0.19, SE = 0.04, *χ*^2^(1) = 14.15, *p* < 0.001; Fig. 1a). Participants maximize the number of correct predictions if they predict approval in 100% of trials for raters in the 87% and 67% groups and if they predict approval in 0% of trials for raters in the 13% and 33% groups. Low self-esteem individuals failed to increase their predictions about being liked for the 67% rater group. In contrast, high self-esteem individuals decrease predictions about being liked for the 33% rater group over time.

We employed a computational modeling approach to gain a deeper insight into the computational mechanisms that underlie these behavioral differences. We fitted a range of computational models to choices and subjective reports of self-worth. We used Bayesian model comparison to determine which model explained participants’ behavior best for the entire dataset while penalizing for increasing complexity (Supplementary Results and Table S2). The winning model explained participants’ choices well, correctly predicting 85% of participants’ choices (95% confidence interval (81–89%); mean pseudo-*r*^2^ = 0.71), and did so equally well for high and low self-esteem participants, *t*(59) = −0.493, *p* = 0.624. In this model SPEs, that express the difference between received and expected feedback, act as teaching signals to simultaneously update expectations about being liked and subjective reports of self-worth.

Expectations about being liked (expected social value, or ESV) were modeled using a Rescorla-Wagner reinforcement learning model^54^:

1 $${\mathrm{ESV}}_k^{t + 1} = {\mathrm{ESV}}_k^t + \eta \;{\mathrm{SPE}}^t$$

where *t* was current trial number, η is a learning rate capturing the weight that participants give to SPEs in updating ESV and *k* indexes the 4 rater groups. We used a softmax function to transform ESVs into action probabilities of predicting to be liked. Initial ESVs for the most positive and the least positive group were estimated using two free parameters and initial ESVs for the other groups were equally spaced in between.

To test if the behavioral tendency to predict being disliked in low self-esteem participants was guided by a lower expectancy of being liked, we compared initial ESV parameter estimates for the two groups. Indeed, participants with low self-esteem had lower initial ESV estimates than those with high self-esteem (Mann–Whitney *U* test, *z* = −2.29, *p* = 0.022), confirming their initial predictions were guided by a lower expectancy of being liked (Fig. 1b). The observation of persistent expectations was reflected in lower learning rates in low self-esteem participants (median = 0.01) compared to high self-esteem participants (median = 0.05; Mann–Whitney *U* test, *z* = −2.30, *p* = 0.021; Fig. 1c). Consistent with our prior work using the same task^14^ learning rates were low, showing that SPEs impact learning about the probability of approval from the four groups relatively slowly. Despite these low learning rates, Bayesian model comparison showed that a model with a learning rate: (1) was preferred over a model without a learning rate (Table S2) and (2) explained choices equally well for high and low self-esteem subjects. The lower learning rates in low self-esteem subjects may explain why their expectations about being disliked are more entrenched than the expectations of subjects with high self-esteem.

We performed two simulation studies to dissociate the impact of global self-esteem on overt behavior (Fig. 1) from an impact on underlying expectations about being liked (Figs. S5, S6). These simulation studies show that in a more “approving” environment (i.e., 75% approval on average) compared to the 50% approval in our experiment, the combination of lower learning rates and lower initial expected social value in low self-esteem participants slows down the development of realistic expectations about being liked (See Supplementary Results). In a more “disapproving environment” (i.e., 25% approval on average), low self-esteem participants have more realistic expectations about social value compared to high self-esteem participants, due to their low initial expectations.

In sum, low self-esteem participants expect to be liked less prior to receiving feedback and manifest a decreased propensity to update their expectations in response to feedback. Specificity in the deficit within the low self-esteem group for learning about the self was confirmed in a control experiment. Here, we showed that high- and low-self-esteem participants had similar expected approval rates and learning rates when they learned about another person’s social value (Supplementary Results). Thus, the behavioral differences support the presence of specific anomalies in how low self-esteem individuals learn about the self rather than a general impairment in social learning.

#### Momentary feelings of self-worth

**Fig. 2:**
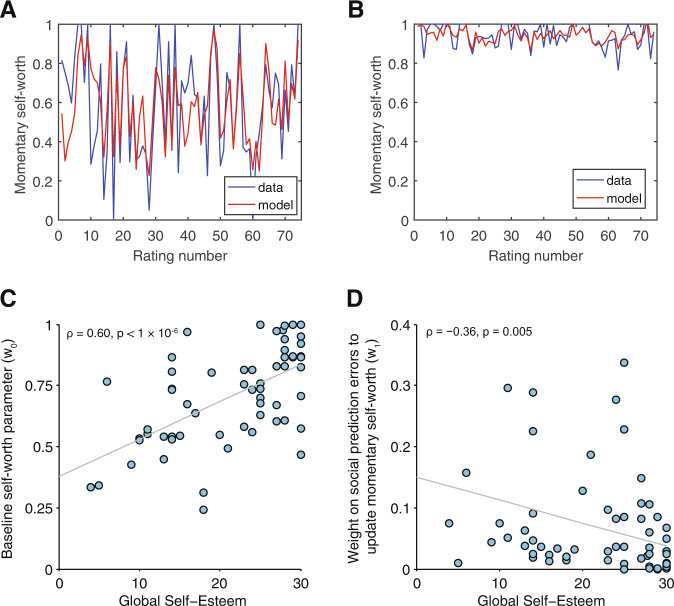
Both baseline momentary self-worth and fluctuations therein depend on global self-esteem. **a** Momentary self-worth ratings (in blue) and predictions of our computational model (in red) in an exemplar participant with low global self-esteem and **b** an exemplar participant with high global self-esteem. **c** People with low global self-esteem had low baseline self-worth parameters *w*_0_ according to the computational model. **d** Subjects with low global self-esteem attributed more weight to social approval prediction errors in determining their momentary self-worth as fitted by computational model parameter *w*_1_.

Low self-esteem participants reported a lower self-worth throughout the task (*M* = 0.62) compared to high self-esteem participants (*M* = 0.80), *t*(59) = −4.07, *p* < 1 × 10^−4^. This group’s self-worth fluctuated to a greater degree (Average SD = 0.12) compared to high self-esteem participants (Average SD = 0.08), *t*(59) = 2.24, *p* = 0.029). We used our computational model to quantify the extent to which momentary self-worth was shaped by social feedback (Fig. 2a, b).

The impact of SPEs on momentary self-worth was captured using an exponential kernel regression model:

2 $${\mathrm{Momentary}}\;{\mathrm{self \hbox{-} worth(t)}} = w_0 + w_1\mathop {\sum}\limits_{j = 1}^t {\gamma ^{t - j}{\mathrm{SPE}}_j + \varepsilon }$$

where *t* was current trial number, *w*_0_ parameterized a ‘baseline’ component of self-worth constant throughout the task, *w*_1_ captured the weight of SPEs on self-worth, and *γ* was a forgetting factor parameterizing a decaying impact of events *j* trials ago. The term *ε* ~ *N*(0, σ) allowed Eq. (2) to serve as a generative model of momentary self-worth by capturing measurement noise (Supplementary Results and Table S2). The model captured changes in momentary self-worth well (mean *r*^2^ = 0.24), and did so equally for people high or low in global self-esteem, *t*(59) = −0.414, *p* = 0.681.

We tested how the two self-esteem groups differed in computational parameters capturing baseline level of self-worth throughout the task (as indexed by parameter w_0_) and the extent to which momentary self-worth depended on social feedback (as indexed by parameter w_1_). A direct comparison between groups yielded a significant difference in baseline level of self-worth (*w*_0_) (Mann–Whitney *U* test, *z* = −3.45, *p* < 0.001), but no evidence of a group difference in dependency of self-worth on social feedback (*w*_1_) (Mann–Whitney *U* test, *z* = −1.54, *p* = 0.123). Recruitment global self-esteem scores (assessed using the Rosenberg self-esteem scale [RSES]) were highly correlated with global self-esteem scores at the time of scanning (*ρ*(59) = 0.74, *p* < 1 × 10^−10^). Exploratory dimensional analyses showed that global self-esteem at the time of scanning was positively associated with *w*_0_ (*ρ*(59) = 0.60, *p* < 1 × 10^−6^; Fig. 2c) and negatively with *w*_1_ (*ρ*(59) = −0.36, *p* = 0.005; Fig. 2d). Together these results indicate participants with high global self-esteem at the time-point they perform the task have a higher baseline self-worth throughout the task compare to those with low self-esteem. Moreover, high self-esteem individuals were relatively more successful in maintaining their self-worth in the face of feedback than low self-esteem individuals where self-worth was more easily perturbed by social feedback. These results were corroborated by model-free analyses (Fig. S4). This suggests that state-like components of global self-esteem captured by the RSES at the time of scanning are better predictors of momentary fluctuations in self-worth than self-esteem’s trait-like components. The w_1_ parameter weights did not correlate with learning rates (*ρ*(59) = −0.07, *p* = 0.613), indicating that the w_1_ parameter and learning rate quantify independent weighting of SPEs in determining distinct self-evaluative beliefs (i.e., “reflected” self-appraisals about being liked vs. “direct” self-appraisals in the form of reported feelings of self-worth). Consistent with this result, simulation studies showed that negative initial expectations and lower learning rates in low self-esteem participants have little effect on self-worth in the task (See Supplementary Results).

#### Self-esteem and interpersonal vulnerability

**Fig. 3:**
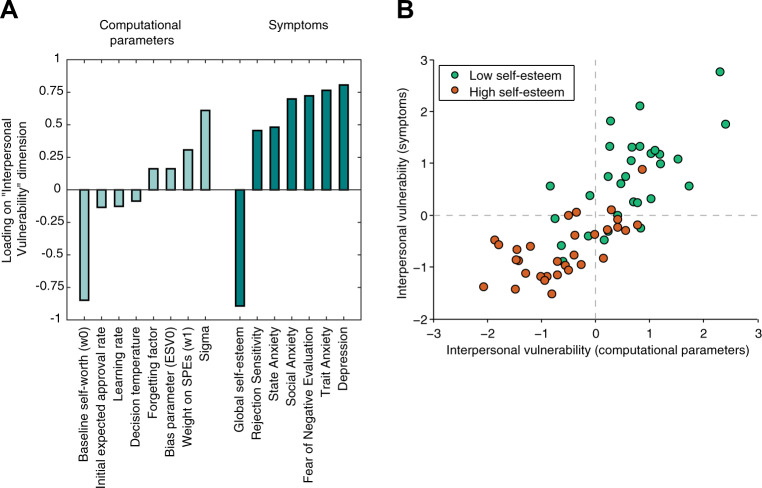
Symptoms of low self-esteem co-vary with computational parameters in a pattern indicative of interpersonal vulnerability. **a** The standardized canonical coefficients for the “interpersonal vulnerability” dimension across computational self-esteem parameters and psychiatric symptoms (*r* = 0.79, *p* < 0.001). **b** Interpersonal vulnerability scores in terms of psychiatric symptoms plotted against interpersonal vulnerability scores in terms of computational parameters with one dot per participant. The high correlation indicates significant co-variation between the two sets of variables captured by the principal CCA dimension (“interpersonal vulnerability”). Higher scores reflect greater interpersonal vulnerability. Canonical correlation in high self-esteem group: *r* = 0.64, *p* < 0.001. Canonical correlation in low self-esteem group: *r* = 0.57, *p* = 0.001.

Participants recruited to have low global self-esteem not only had lower global self-esteem than high self-esteem participants on the day of scanning (*p* < 1 × 10^−9^), but they also scored higher on self-report measures of interpersonal sensitivity (all *p*s < 1 × 10^−4^), anxiety (all *p*s < 0.016) and depression (*p* < 1 × 10^−5^; Table 1). To best describe behavioral variation in our sample, and to simultaneously characterize the dimensionality of psychopathology and behavior, we implemented a CCA over both self-reported psychiatric symptoms and computational self-esteem parameters^55^. The CCA yielded one significant canonical dimension (Wilks’s *λ* = 0.15, F(56,253) = 1.76, *p* < 0.001), which had a canonical correlation of 0.79 between symptoms and computational parameters. Global self-esteem made the greatest contribution to the canonical dimension (Fig. 3a). The constellation of positive and negative associations on this canonical dimension generally replicated the constellation of loadings on a dimension of “interpersonal vulnerability” we identified in previous work^14^. Symptoms of interpersonal sensitivity (e.g. rejection sensitivity and fear of negative evaluation), anxiety, and depressed mood and weight on SPEs (*w*_1_) were positively related to interpersonal vulnerability. Global self-esteem, baseline self-worth in the social evaluation task (*w*_0_) and initial expected approval rate were negatively associated with “interpersonal vulnerability” (Fig. 3a).

Participants in the low self-esteem group significantly scored higher on vulnerability in terms of computational self-esteem parameters (*t*(59) = 5.88, *p* < 1 × 10^−6^) and in terms of psychiatric symptoms (*t*(59) = 7.35, *p* < 1 × 10^−9^) than those in the high self-esteem group. Despite these group differences considerable variation remained in behavior and symptoms within groups. To test whether variation within groups mapped onto the interpersonal variability dimension identified across groups, we correlated variation in parameters to variation in symptoms for each group separately. These analyses showed those in the high self-esteem group who reported more symptoms than other high self-esteem participants, had elevated loadings for computational parameters indicative of vulnerability (canonical correlation within high self-esteem group, *r* = 0.64, *p* < 0.001). Similarly, those in the low self-esteem group who reported fewer symptoms than other low self-esteem participants, had lower loadings for computational parameters indicative of vulnerability (canonical correlation within low self-esteem group, *r* = 0.57, *p* < 0.001; Fig. 3b). These analyses suggest that individual variation in symptoms can be explained by specific computational parameters, which cluster on a dimensionally arrayed marker of vulnerability that is present across the entire self-esteem spectrum.

### Neuroimaging results

#### Neural signatures of expectations about being liked

**Fig. 4:**
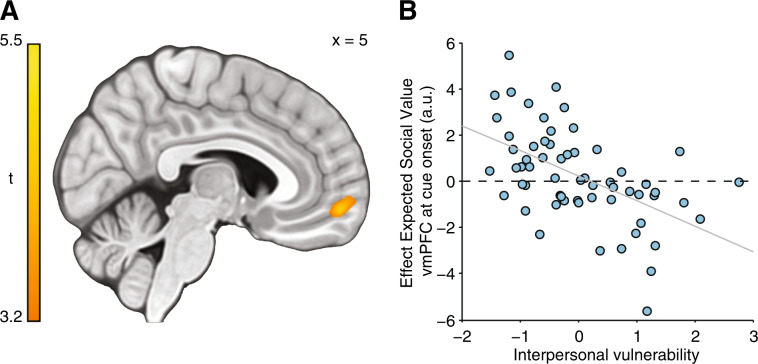
Neural signatures of expected social value in vmPFC are modulated by individual differences on a dimension of interpersonal vulnerability. **a** A whole-brain analysis testing for an interaction between interpersonal vulnerability and expected social value (derived from computational modeling) at cue-onset across the two self-esteem groups revealed a significant cluster in vmPFC, thresholded at *p* < 0.05 (FWE cluster-corrected using a cluster-forming threshold of *p* < 0.001). **b** To visualize the interaction between interpersonal vulnerability and vmPFC responses to expected social value, we plotted average BOLD responses to expected social value extracted from the functional ROI shown in **a** against interpersonal vulnerability scores. Regression line plotted for illustration purposes only. *n* = 61 subjects.

To examine neural signatures of expectations about being liked, we constructed a GLM to identify brain activity, at cue onset, that varied parametrically with ESV (derived from our computational model). We found no evidence for a main effect of ESV across the whole sample, collapsing across both self-esteem groups, nor differences between the high and low self-esteem groups (using a whole-brain independent samples t-test) that survived correction for multiple comparisons. However, a whole-brain analysis testing for an interaction between interpersonal vulnerability and ESV, using subject-specific scores on the ‘interpersonal vulnerability’ dimension as a between-subjects regressor, revealed a cluster in vmPFC (Fig. 4; peak coordinates (−2,59, −11; *t*(59) = 4.96, *Z* = 4.52, *k* = 687, *p* = 0.005, FWE cluster-corrected). This indicates that when making predictions about being liked, more vulnerable participants on an interpersonal vulnerability dimension have an attenuated ESV signal in vmPFC compared to those ranked as less vulnerable. We replicated our prior work showing that SPEs correlated with activity in ventral striatum/sgACC activity (*z* = 2.95, *p* = 0.003). We found no evidence for a difference in neural processing of SPEs between low and high self-esteem participants (Mann–Whitney *U* test, *z* = 0.64, *p* = 0.521; Fig. S8). Interpersonal vulnerability did not significantly correlate with SPE-related activity in ventral striatum/sgACC (*ρ*(59) = 0.23, *p* = 0.073) or anterior insula (*ρ*(59) = 0.08, *p* = 0.534).

#### Neural signatures of updates in momentary self-worth

**Fig. 5:**
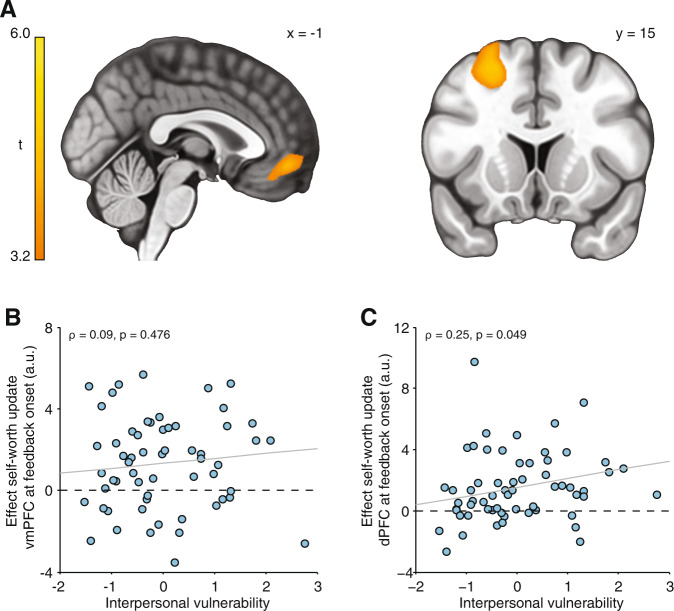
Neural signatures of feedback-induced updates in momentary self-worth. **a** Updates in momentary self-worth upon receipt of feedback are tracked within vmPFC and left dorsal prefrontal across both self-esteem groups, thresholded at *p* < 0.05 (FWE cluster-corrected using a cluster-forming threshold of *p* < 0.001). **b** Updating-related activity extracted from the functional vmPFC region of interest (mean BOLD response across entire functional ROI shown in the sagittal plane in **a**; 3290 mm^3^) did not correlate with interpersonal vulnerability (*ρ*(59) = 0.09, *p* = 0.476), **c** Updating-related activity extracted from the functional dPFC region of interest (mean BOLD response across entire functional ROI shown in the coronal plane in **a**; 9730 mm^3^) correlated positively with interpersonal vulnerability (*ρ*(59) = 0.25, *p* = 0.049). *n* = 61 subjects.

To examine neural signatures of feedback-induced updates in momentary self-worth, we constructed a GLM to identify regions responding parametrically to trial-by-trial updates in self-worth at the moment of feedback presentation (derived from our computational model). A whole-brain independent samples *t*-test testing for group differences between high and low self-esteem participants did not identify any cluster that survived correction for multiple comparisons. To explore whether a dimensional marker of vulnerability better captured inter-individual differences in updating-related brain activity, we first performed a whole-brain collapsing across both self-esteem groups to identify brain regions functionally involved in self-worth updates. This analysis revealed significant clusters within vmPFC (peak coordinates: −3,47, −11, *t*(60) = 4.37; *Z* = 4.06, *k* = 584, *p* = 0.01, FWE cluster-corrected) and left dorsal prefrontal cortex (dPFC; in Brodmann Area [BA] 8 m (peak coordinates: −23,29,51, *t*(60) = 5.95; *Z* = 5.25, *k* = 2896, *p* < 1 × 10^−7^, FWE cluster-corrected; Fig. 5a). Next, we extracted activity from the two clusters identified in the whole-brain analysis and correlated it against subject-specific scores on the ‘interpersonal vulnerability’ dimension. These analyses revealed a significant positive association between activity in dPFC and interpersonal vulnerability (*ρ*(59) = 0.25, *p* = 0.049; Fig. 5c), but no association between updating-related activity in vmPFC and interpersonal vulnerability (*ρ*(59) = 0.09, *p* = 0.476; Fig. 5b).

## Discussion

This study shows that participants with low self-esteem have a reduced tendency to use social feedback to learn how much they are liked by others, coupled with an enhanced tendency to use social feedback in determining subjective reports of self-worth. Computational modeling revealed these individual differences arise out of differential weighting of SPEs in updating exectations about being liked, compared to feelings of self-worth. This dissociation between expectations about being liked and feelings of self-worth was paralleled at a neural level and this became especially clear upon taking a dimensional approach. Low global self-esteem made the greatest contribution to a canonical dimension of interpersonal vulnerability characterized by interpersonal difficulties, symptoms of depression and anxiety, and amplified computational self-esteem parameters. Participants who scored higher on this dimension of vulnerability showed a blunted neural expression of expected social value in ventromedial PFC when determining whether others will like them, and heightened dorsal PFC activity that co-varied with fluctuations in self-worth when finding out whether others actually liked them.

A traditional framework for understanding low self-esteem derives from the notion that people with low self-esteem have acquired a persistent belief that others will not approve of them based on past negative appraisals by others^3,11,13^. Our results show that impairments in a reinforcement-learning mechanism can explain how such negative “reflected self-appraisals” are maintained. The key observation here is that while participants with high and low self-esteem express indistinguishable SPE signals in VS/sgACC, low self-esteem participants are slower to update their, already lower, estimate of social value in response to these SPEs. Although low self-esteem participants generally predicted they would be disliked more often, the contrast with high self-esteem participants was most prominent in predictions about raters in the mildly positive 67% group. This is consistent with observations showing that people with low self-esteem are more likely to expect rejection and feel undeserving of acceptance^36,56,57^. However, low self-esteem did not significantly impact on predictions about being liked by the unambiguously positive 87% group. Speculatively, we suggest beliefs about being disliked may be more pronounced in situations carrying greater levels of uncertainty or ambiguity.

Our neuroimaging results indicate that a “reflected” self-value signal (i.e., ESV, or “how positive do others view me”) used to predict whether others like us is computed in a region in anterior vmPFC. This dovetails with animal and human studies showing that neurons in vmPFC perform both self-referential and social value computations^4,52,58–61^. Critically, interpersonal vulnerability modulated ESV-related activity in vmPFC, suggesting that vulnerability in terms of low self-esteem, and co-occurring symptoms, bias formation of value-representations needed to infer whether others like us. The region we found overlaps with an anterior subportion of vmPFC activated in those who continue to see themselves as socially desirable under threat of social rejection^25^. Our results further showed that an adjacent subregion in vmPFC tracked updates in self-worth at the moment of feedback delivery. These “direct” self-value-signals in vmPFC were not modulated by global self-esteem or vulnerability. Together these findings indicate that neighboring subregions in vmPFC encode self-value signals at distinct time points (cue vs. feedback), and are involved in self-appraisal processes (reflected vs. direct) subject to differential modulation by a self-esteem related vulnerability.

While update-related activity in vmPFC did not vary as a function of interpersonal vulnerability, this measure positively impacted on activity in dPFC (BA 8 m) during updates in self-worth. Activity in BA 8 m increased with boosts in momentary self-worth, an effect amplified in those who were more vulnerable. This correlation was not observed in our previous study^14^, most likely because of limited variance in global self-esteem and psychiatric symptoms. This may also explain why vulnerability did not correlate with SPE-related activity in the insular region we found to correlate with interpersonal vulnerability in a sample with average self-esteem^14^. Unlike neighboring regions in frontal eye fields or premotor cortex, BA 8 m is functionally coupled with vmPFC^62^ and consistently co-activates with vmPFC when receiving social approval^63,64^ or during an encoding of subjective value^21^ (Fig. S9). The specific location of the cluster in BA 8 m we identified overlaps a region where activity during positive emotion regulation monotonically increased with improvements in positive affect^65^. We speculate this subregion of dPFC may contribute to a boost in positive feelings in response to social approval, particularly in vulnerable individuals who show a greater dependence on social approval for their self-worth. Given we had no a priori hypothesis about this region and that the effect was not robust to excluding participants from our analyses who reported doubts about the cover story, this result should be interpreted with caution and needs replication in larger samples.

Self-esteem is not an independent disposition, but belongs to an organized structure of psychological characteristics that predict mental health^28^. Our findings reveal computational signatures of learning about the self in an ecologically valid sample and only allow limited claims about the specificity of self-esteem to the mechanisms identified. Translational importance was demonstrated by analyses showing that symptoms that accompany low self-esteem (e.g. anxiety and low mood) co-vary with computational self-esteem parameters in a pattern suggestive of interpersonal vulnerability. Strikingly, this dimension cut across self-esteem groups showing that high self-esteem individuals who reported more symptoms along this dimension had amplified computational parameters akin to individuals with low self-esteem. This dimensional perspective was corroborated by our neuroimaging findings showing individual variation in neural processing was better explained by differences along a continuous dimension of vulnerability rather than coarse group differences in self-esteem. A lack of group differences on a neural level may also reflect the possibility that neural differences between groups may have been too small to detect within a sample size of 61 participants.

Our results can help resolve a puzzling observation that low self-esteem is characterized by both a stable negative view of the self and greater instability in self-esteem^5^. Using computational modeling, we show that this apparent paradox is explained by low self-esteem participants underweighting SPEs when learning what to expect from others, and overweighting these learning signals when updating feelings of self-worth. Slow updating of social value was associated with persistent expectations about being disliked, while fast updating of subjective self-worth was associated with greater instability in self-esteem. Our computational framework into the neural underpinnings of learning from social feedback in participants at the extreme ends of a self-esteem distribution hints at neurobiological mechanisms of vulnerability for mental illness.

## Supplementary information

Supplementary Material

## Data Availability

Analysis scripts are available upon request by contacting the corresponding author.
