# Supplementary material for: Neurocomputational mechanisms underpinning aberrant social learning in young adults with low self-esteem
Source: Transl Psychiatry. 2020 Mar 17;10:96. doi: 10.1038/s41398-020-0702-4 (PMC7078312; doi:10.1038/s41398-020-0702-4)
Supplement: Supplementary file 1 — Supplementary Material [file 41398_2020_702_MOESM1_ESM.docx]

**Supplementary Material**

**Neurocomputational mechanisms underpinning aberrant social learning in young adults with low self-esteem**

Geert-Jan Will^1,2,3*^, Michael Moutoussis^1,2*^, Palee M. Womack^2^, Edward T. Bullmore^4,5,6^, Ian M. Goodyer^4,5^, Peter Fonagy^7^, Peter B. Jones^4,5^, NSPN Consortium, Robb B. Rutledge^1,2^, & Raymond J. Dolan^1,2^

**Affiliations:**

^1^ Max Planck University College London Centre for Computational Psychiatry and Ageing Research, London, UK

^2^ Wellcome Centre for Human Neuroimaging, University College London, UK

^3^ Institute of Psychology, Leiden University, Leiden, The Netherlands

^4^ Department of Psychiatry, University of Cambridge, Cambridge, United Kingdom

^5^ Cambridgeshire and Peterborough National Health Service Foundation Trust, Cambridge, UK

^6^ ImmunoPsychiatry, GlaxoSmithKline Research and Development, Stevenage, UK

^7^ Research Department of Clinical, Educational and Health Psychology, University College London, London, UK

* Shared first authorship

**Corresponding author:** Geert-Jan Will, Institute of Psychology, Leiden University, Wassenaarseweg 52, 2333 AK Leiden, The Netherlands. Phone: +31 71 527 3670. Email: [gjwill@gmail.com](mailto:g.j.will@fsw.leidenuniv.nl)

**This PDF file includes:**

Supplementary methods

Supplementary results

Figs. S1 to S9

Tables S1 to S2

Supplementary references

**Supplementary Methods**


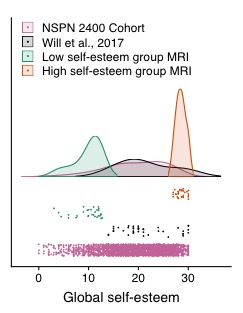


**Fig. S1.** **Distributions of global self-esteem.** *Participants were drawn from a large representative community sample (n=2,402) based on longitudinal assessments of global self-esteem (average Rosenberg self-esteem scores across assessments in pink).* *Mean self-esteem of the large sample was 19.7 (on a scale of 0 to 30; SD=5.62; bottom decile=0-12 and top decile=27-30). We selected for further study, from this large sample, 30 low self-esteem participants (recruitment Rosenberg self-esteem score in green) and 31 high self-esteem participants (recruitment Rosenberg self-esteem score in orange) from the bottom and top decile of self-esteem scores respectively We plot the distribution of Rosenberg self-esteem scores in our previous study (n=40; in black)^1^ to illustrate* *individual variation obtained when sampling randomly from the population. Figures created using code for ‘Raincloud plots’^2^.*

**
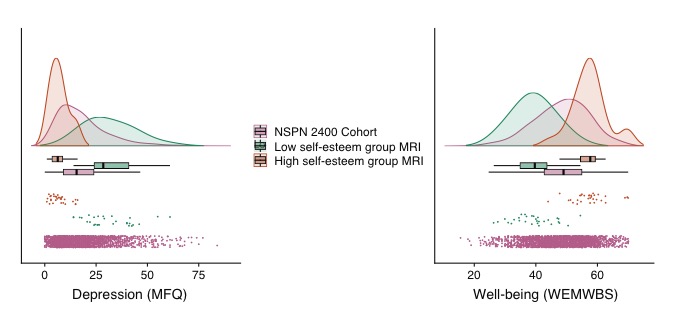
**

**Fig. S2. Distributions of depression and well-being in the NSPN 2400 cohort and in high and low self-esteem participants in the present study.** *We recruited individuals with low self-esteem and individuals with high self-esteem to maximize the contrast between those with substantial subclinical self-esteem related symptoms and those who experience very few symptoms. Global self-esteem (assessed using the Rosenberg self-esteem scale) in the large cohort (n=2,402) strongly correlated negatively with depression (assessed using the Mood and Feelings Questionnaire [MFQ], r=-.79, p<1×10^-5^) and positively with well-being (assessed using the Warwick-Edinburgh Mental Well-being Scale [WEMWBS], r=.80, p<1×10^-5^). Left panel: Depression severity (averaged over 1-3 measurements) in the two MRI groups relative to population: low self-esteem group: 78.5 ± 33.3 percentile and high self-esteem group: 16.4 ± 6.1). Right panel: Well-being (averaged over 1-3 measurements) level in the two MRI groups relative to population: low self-esteem group: 16.4 ± 0.04 percentile, high self-esteem group: 85 ± 0.04 percentile). Figures created using code for ‘Raincloud plots’^2^.*

**Social Evaluation Task**

Participants performed a social evaluation task in an MRI scanner. In this task they received approval or disapproval feedback from peers who had seemingly viewed an online character profile. Participants created this online profile at least 5 days (mean=19.6, SD=21.4) before performing the task in the scanner (see Table S1 for the instructions participants received for creating their profile).

**Table S1. Instructions for creating online profile.**

| **Instructions and questions “Create your Profile”** |
| --- |
| You are now going to create a profile about yourself. You will create your profile via this online platform, so that we can show it to other people taking part in the study. These people are men and women between the ages of 18 and 30. They will be asked what they think about you. They can choose to like or dislike you.  This is how they are asked make their decision:    “Do you like this person? Do you think you could you be friends with them in real life? Or do you think this person is boring and are you not interested in getting to know them any better?  Press the “like” button, if you think you could be friends with this person in real life. Press the “dislike” button if you are not interested in getting to know them.”    Your profile will be made up of your answers to 5 personal questions. Together they will give others a good idea about who you are. Please answer each question thoughtfully and honestly in roughly 2-3 sentences. Take the time to answer, because this is the only information that others will have to judge you.  Questions:  1. If we asked your friends and family about your best qualities, what might they say?  2. And what would they say were your worst qualities?  3. What are you most afraid of?  4. My favorite things in life are:  5. I really dislike people who (for example people who are lazy, mean to others, or make annoying noises when they eat): |

In the scanner participants received feedback from 184 independent raters. Before performing the task participants were told that raters were ordered into 4 groups based on the number of profiles they had evaluated positively. Unbeknownst to the participants, social feedback was generated by an algorithm. This resulted in receipt of approval feedback on 92 trials and disapproval feedback on the other 92. The probability of receiving approval feedback depended on the rater’s group membership, with positive feedback in 87%, 67%, 33%, and 13% of the trials, spanning the first to fourth quartile rater groups respectively.

On each trial, participants were presented the name of a rater and a color cue that indicated which of the 4 groups this rater belonged to (Fig. S3). Participants had 3 seconds to indicate whether they expected to be liked by this specific rater. After a 6 second delay, they received either approval (“a thumbs up symbol”) or disapproval feedback (“a thumbs down symbol”) for 1 second. After every 2-3 of such feedback trials, participants were presented with a self-esteem probe in the form of the question “How good do you feel about yourself right at this moment?” for 4 s. Subsequently, they had 4 seconds to move a cursor along a visual analog scale with endpoints ‘very bad’ and ‘very good’. The color cues were randomized in blocks of four.

**Fig. S3. Design social evaluation task.** *A)* *Participants received approval or disapproval feedback from 184 peers (or raters) who had seemingly viewed their online character profile. These raters were sorted into four groups based on their overall propensity to give approval feedback. Participants received approval feedback in 87%, 67%, 33%, and 13% of the trials depending on the rater’s group. B) On each trial participants were presented with a name of a new rater and a color cue indicating which group a liked by this rater. After a delay of 6 seconds, participants received feedback in the form of either a thumbs-up or a thumbs-down symbol (displayed for 1 second) indicating approval or disapproval respectively. After every 2-3 choice trials, participants reported their momentary feelings of self-worth (total of 75 ratings).*

If participants failed to make a prediction within 3 seconds a “too late” screen was displayed for 1.5 seconds. Prior to scanning they were informed that if this happened £0.50 would be subtracted from a potential endowment they would play with in a separate task (Dictator Game; see Fig. S7). Monetary losses were independent of participants’ propensity to predict being liked and the actual feedback they received, so that payment did not depend on participants’ accuracy. Missed trials were rare (median=1) and were excluded from further analyses. The task was administered in three blocks, which lasted about 17 minutes each. For 2 participants the projector screen went blank for a part of a block. We excluded data collected during these intervals, but analyzed their remaining data. At the end of each block, participants received performance feedback about how many trials they correctly predicted in order to increase engagement with the task. After the experiment, participants filled out funneling suspicion probe to assess whether participants believed the feedback was derived from actual appraisals from other people. This questionnaire consisted of the following three open-ended questions: 1) What did you think of the task? What was your overall experience?; 2) How did you like that other people on the internet liked/disliked you based on personal information?; 3) What do you think this study was about?

**fMRI Data Acquisition and Analysis**

We collected fMRI data in 3 runs (median volumes=1126; range=1091-1147; total number of volumes acquired varied depending on participants’ choice times). Each volume was acquired with an ascending slice acquisition order in 40 slices with the following specifics: repetition time (TR)=2.8 sec, echo time (TE)=30ms, slice thickness=2 mm, slice gap=1 mm gap, slice tilt of −30° (T>C), field of View=192 × 192 mm^2^. The first five volumes from each run were discarded to account for T1-saturation effects. To correct the EPIs for field-strength inhomogeneities, we acquired field maps (TE=10 and 12.46 ms, TR=102 ms, matrix size 64 x 64, with 64 slices, voxel size=3 mm^3^).

After the EPIs were collected, we acquired structural MRI data using quantitative multiparameter maps (MPMs) in a 3D multiecho fast low-angle shot (FLASH) sequence with a resolution of 1-mm isotropic voxels^3^. This protocol consisted of three flash sequences and Three FLASH sequences with 1 mm isotropic resolution were acquired with different weightings depending on choice of repetition time (TR) and flip angle (α): 1) predominantly T1-weighted (TR/α=18.7 ms/ 20°), 2) predominantly proton density-weighted (TR/α=23.7 ms/6°), and 3) MT-weighted (TR/α=23.7 ms/6°) with a 2 kHz off-resonance RF pulse (4ms duration and 220 degree nominal flip angle) applied prior to excitation. Multiple gradient echoes were acquired with alternating readout polarity at six equidistant echo times (TE) from 2.2 to 14.7 ms for the T1-weighted MT-weighted acquisitions and at 8 equidistant TE from 2.2 ms and 19.7 ms for the PDw acquisition. To account for inter-scan head motion, we corrected for differential receive field modulation between scans using sensitivity maps^4^ before calculating the quantitative maps.

**Supplementary Results**

**Analysis of variance on self-reported feelings of self-worth in response to feedback**

We performed a repeated-measures analysis of variance (ANOVA) on self-reported feelings of self-worth with global self-esteem (2 levels: high vs. low) as between-subjects factor and social feedback (2 levels: approval vs. disapproval) and rater group (4 levels: 87%, 67%, 33% and 13% approval) as within-subjects factors. This analysis revealed significant main effects of global self-esteem (F(1,59)=16.29, p<.001, η_p_^2^=0.22), social feedback (F(1,177)=36.40, p<1×10^-6^, η_p_^2^=0.38) and rater group (F(3,177)=14.87, p<1×10^-7^, η_p_^2^=0.20). There was a significant two-way interaction effect between social feedback and rater group, F(3,177)=4.46, p=.005, η_p_^2^=0.07 and a significant three-way interaction between global self-esteem, social feedback, and rater group, F(3,177)=2.81, p=.041, η_p_^2^=0.05.

Follow-up comparisons showed that participants with high self-esteem reported higher self-worth across conditions (see Fig. S4). Self-worth was higher after approval than after disapproval from all 4 rater groups (all Fs > 14.45, all ps<.001). Self-worth was highest after approval from the most negative rater group (13% group, i.e. most unexpected approval) compared to the other 3 rater groups (all ps<.023), and lowest after disapproval from the positive groups (87% and 67% group, i.e., unexpected disapproval) compared to the other 2 rater groups (all ps<1×10^-4^). Follow-up analyses on the three-way interaction effect showed that ‘rater group’ interacted with global self-esteem for approval feedback (p=0.032), but not disapproval, feedback (p=.221). High self-esteem participants reported a higher level of self-worth after approval from all rater groups (no effect of rater group, p=.305), while low self-esteem participants reported higher self-worth after approval from the most negative group (13% group, i.e., most unexpected approval) compared to the other 3 rater groups (all ps<.021). After disapproval feedback, both self-esteem groups showed a similar pattern, with self-worth lowest after disapproval from the positive rater groups (87% and 67% groups, i.e., unexpected disapproval) compared to negative rater groups (33% and 13% groups), all ps<023). Together these results are consistent with our computational modeling-based analyses showing that 1) low self-esteem participants have lower baseline self-worth throughout the task and 2) the impact of social feedback on self-worth is moderated by expectations about feedback (here indirectly assessed using rater group as an index of approval probability) and global self-esteem.

**Fig. S4. Subjective self-worth in response to approval and disapproval feedback in low (panel A) and high self-esteem (panel B) participants.** *Low self-esteem participants reported lower self-worth throughout the task (main effect global self-esteem, p<.001). In both self-esteem groups, self-worth was higher after approval than after disapproval feedback from all 4 rater groups (all ps<.001). High self-esteem participants reported similar high levels of self-worth after approval from all rater groups (no effect rater group, p=.305), while low self-esteem participants had higher self-worth after approval from the most negative group (13% group) compared to the other 3 rater groups (all ps<.021). Following disapproval feedback, both self-esteem groups showed a similar response pattern where self-worth was lowest after disapproval from raters from positive groups (87% and 67% groups) compared to rater from negative groups (33% and 13% groups), all ps<.023. Data are represented as mean ± SEM.*

**Model comparison: Computational models of momentary self-worth**

We used maximum log-likelihood fitting with flat priors over the parameters to fit a range of computational models to individual subjects’ behavioral data. We considered the summed log likelihood of the model prediction over choices (i.e. participants’ predictions about being liked) and the summed log density of the model prediction over subjective reports of momentary self-worth. We compared a range of computational models in terms of how well they explained the data, while penalizing for complexity (i.e. a larger number of parameters) using the Bayesian Information Criterion (BIC). Results of Bayesian model comparison are reported in Table S2. We used the best fitting model (model 1) to compare parameters between the two self-esteem groups.

In model 1 subjective reports of self-worth are modeled as follows:

$\text{Momentary self-worth(t)}=w_{0}+w_{1}\sum_{j=1}^{t} \gamma^{t-j}SPE_{j}+\varepsilon$ (Equation S1)

where t and j are trial numbers, w0 is a baseline self-worth parameter, w1 is a parameter capturing the extent to which momentary changes in self-worth are shaped by social approval prediction errors (SPE) and γ is a forgetting factor (0≤γ ≤ 1) that allowed the influence of past prediction errors to decay over time. The term ε ~ N(0, σ) allowed equation 1 to serve as a generative model of self-worth by capturing measurement noise. Social prediction errors are teaching signals that represent the difference between expected and received social feedback:

$SPE^{t}=Social\text{ }feedback-ESV^{t}$ (Equation S2)

where social feedback was 1 for approval and -1 for disapproval. Expected social feedback (or expected social value; ESV) was estimated using a reinforcement-learning model fitted to participants’ choice behavior:

$ESV_{k}^{t+1}=ESV_{k}^{t}+\eta\text{ }SPE^{t}$ (Equation S3)

where η is a learning rate (1 × 10^-5^  ≤ η ≤ 1) that quantifies the degree to which subjects update their expected social value (ESV) for each rater group k in response to social approval prediction errors (SPE). Initial ESV values were fitted using two free parameters indexing initial expected approval rates for the most positive (i.e., and the least positive group (i.e., ). Starting values for the other groups were equally spaced in between those 2 values. Expected social value was transformed into an action probability of predicting social approval as follows:

$\pi_{L}=\frac{1}{1+e^{\frac{-(ESV+ESV_{0})}{T}}}$ (Equation S4)

where T is a decision temperature parameter and ESV_0_ is a response bias parameter, which quantifies the value of predicting to be liked over and above estimated expected social value.

Model 2 is identical to model 1, but does not include a response bias parameter ESV_0_.

Model 3 assumes participants begin the task with the correct expectations for each of the four rater groups (87%, 67%, 33% and 13%) and that they do not update their expected social value based on the feedback they receive. This model thus omits the 2 parameters for initially expected approval rates and the learning rate parameter η. Model 3 was compared against model 1 to establish that participants indeed dynamically update their estimates of expected social value through learning from feedback despite having learned the rank ordering of the 4 groups prior to performing the task.

Model 4 had a separate expectations term in the self-worth equation:

$\text{Momentary self-worth(t)}=w_{0}+w_{1}\sum_{j=1}^{t} \gamma^{t-j}SPE_{j}+w_{2}\sum_{j=1}^{t} \gamma^{t-j}ESV_{j}+\varepsilon$ (Equation S5)

To test the hypothesis that momentary self-worth fluctuates in response to social prediction errors depend on the outcome of expectations about social feedback rather than the valence of feedback, we compared two additional models.

Model 6 assumes that every instance of positive (or negative) social feedback (SF) has a similar impact.

$\text{Momentary self-worth(t)}=w_{0}+w_{1}\sum_{j=1}^{t} \gamma^{t-j}SF_{j}+\varepsilon$ (Equation S6)

We hypothesized that such a model without expectations is likely to provide a worse fit for participants’ choice behavior given that their choices were strongly predicted by group membership of the raters (χ^2^(3)=5883.47, p<1 × 10^-15^). Therefore, we fitted this model only to subjective reports of momentary self-worth and compared it to model 5. Like model 1, model 5 includes 2 free parameters capturing differences in expectations across the rater groups.

We computed the BIC for each model as follows: , where n is the number of choices and self esteem ratings used to compute the likelihood, k is the number of fitted parameters and $\hat{L}$ is the maximized value of the likelihood function of the model (i.e., the summed log likelihood of the model prediction over the predictions and the summed log density of the model prediction over the subjective self-worth ratings). For model comparison, BIC measures were summed across participants and the model with the lowest BIC was preferred.

**Table S2. Comparisons of fits of computational self-esteem models.**

| **Model** | k | Mean pseudo-r^2^ choices | Mean r^2^ self-worth ratings | BIC | BIC-BIC_model1_ |
| --- | --- | --- | --- | --- | --- |
| **1: Learning and positive bias** | **9** | **0.71** | **0.24** | **-1693** | **0** |
| 2: Learning, but no bias | 8 | 0.64 | 0.23 | -909 | 428 |
| 3: Correct initial beliefs about approval | 6 | 0.67 | 0.20 | -1646 | 47 |
| 4: Separate term for expectations | 10 | 0.70 | 0.25 | -1130 | 563 |

| **Model** | k | Mean r^2^ self-worth ratings | BIC | BIC-BIC_model1_ |
| --- | --- | --- | --- | --- |
| **5: Free initial beliefs about approval** | **6** | **0.24** | **-9602** | **0** |
| 6: Outcome valence only | 4 | 0.23 | -9274 | 328 |

k is the number of fitted parameters. Bayesian Information Criterion (BIC) measures are summed across all participants. Lower BIC values indicate a more parsimonious model fit. Mean squared error over subjective self-worth ratings indicates goodness of fit.

**Simulation study 1**

We used our computational model to generate expected social values for the four “rater groups” as they evolved during the experiment. We sampled each participant 100 times to average over irrelevant sources of variation (e.g., order of stimulus presentation), leading to a sample of 6100 simulated participants. We endowed participants in both self-esteem groups with either the median fitted learning rate of their “native” group (Fig. S5A) or the “opposite” group (Fig. S5B). Endowing high self-esteem participants with a median learning rate from low self-esteem participants (i.e., a lower learning rate) made their expected social value higher. Endowing the low self-esteem people with a median learning rate from high self-esteem participants (i.e., a higher learning rate) made them update their expected social value more properly to align with the approval rates for all four rater groups.


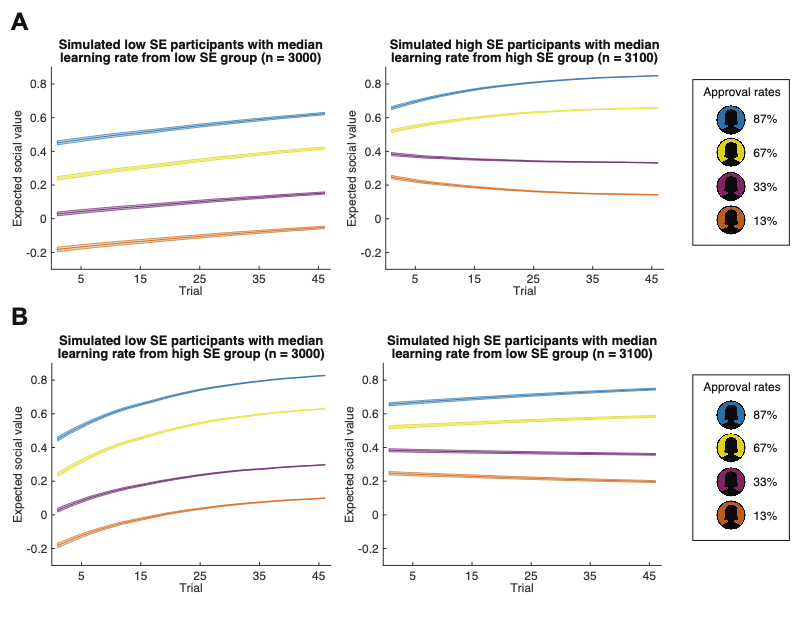


**Fig. S5. Simulations of expected social value for low and high self-esteem participants.** A) Simulations of expected value keeping all parameters at their fitted values and using median learning rates of participants’ “native” group. B) Simulations of expected value keeping all parameters at their fitted values and using median learning rates of participants’ “opposite” group. These simulations show that the low self-esteem group would develop more realistic expectations if they had learning rates akin to those of high self-esteem participants. Shaded areas represent standard error of the mean.

**Simulation study 2**

In the real world, people sort themselves into groups that are likely to give more positive than negative feedback. In that sense, our experiment with an overall 50% approval rate was harsh. We ran two further simulations where we expose simulated participants to more positive raters (overall approval rate=75%; with four rater groups giving 91%, 80%, 70%, and 59% approval feedback) and more negative raters (overall approval rate=25%; with four rater groups giving 41%, 30%, 20%, and 9% approval feedback). We endowed participants with a median fitted learning rate of their native group.

When exposed to more positive raters, low self-esteem participants persist much longer with unrealistically pessimistic expectations compared to high self-esteem participants (Fig. S6A). They do not properly update their expectations to make them realistic in terms of that of positive raters. When exposed to more negative raters, expectations about being liked of the low self-esteem participants are more realistic than those with high self-esteem (Fig. S6B). Despite not properly updating their expectations about being liked in response to more positive raters, low self-esteem participants benefit from more approval in terms of their momentary self-worth (average 10% increase in self-worth [from 0.65 to 0.74 on a scale of 0 to 1] compared to 50% approval rate).


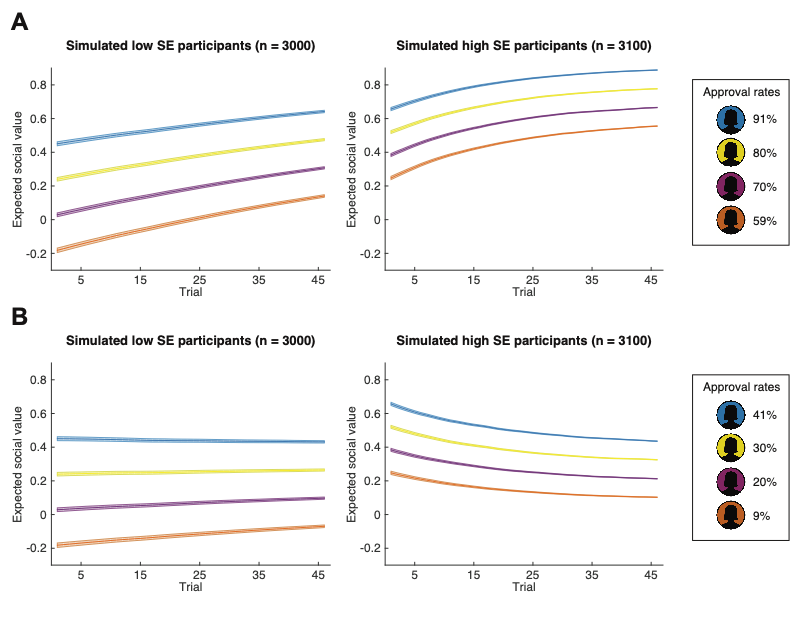


**Fig. S6. Simulations of expected social value for low and high self-esteem participants exposed to more positive or negative raters.** A) In response to more positive raters than in our experiment (overall approval rate=75%; with four rater groups giving 91%, 80%, 70%, and 59% approval feedback), low self-esteem participants persist with unrealistic pessimistic expectations compared to the high self-esteem participants. B) In response to more negative raters than in our experiment (overall approval rate=24%; with four rater groups giving 41%, 30%, 20%, and 9% approval feedback), low self-esteem have more realistic expected social value due to their initial negative expectations. Shaded areas represent standard error of the mean.

**Differences between high and low self-esteem participants are specific to self-evaluation**

To rule out the possibility that the behavioral differences in the fMRI task are caused by general impairments in (social) learning, participants performed an “other evaluation” task. This task was identical to the task performed in the scanner except that participants were not objects of evaluation themselves, but predicted whether another participant (of the same gender and age) was liked and then observed the feedback the other person received. They made 64 predictions (about feedback from 16 raters in each group) and reported on their self-worth after every 2-3 trials (total of 27 ratings).

Participants correctly predicted the feedback in 71% of the time (which approached the optimum of 76%) and predicted that the other person would be liked 50.8% of the time. Choice patterns were reliably predicted by the same Rescorla-Wagner reinforcement-learning model^5^ used to fit participant’s choice behavior when they were the object of evaluation themselves. The model explained choice behavior well, correctly predicting 84% of participants’ choices (95% confidence interval (81–90%); mean pseudo-r2=0.72), and did so equally well for participants in the high or low self-esteem groups, t(59)=-.670, p=.231.

Unlike when the self was the object of evaluation, no differences were found between high and low self-esteem participants in frequency of predicting that the other person would be liked (t(59)=1.14, p=.257), initial expected approval rates (Mann-Whitney U test, z=-0.99, p=.322) or learning rates (Mann-Whitney U test, z=-0.63, p=.528). In contrast to when the self was evaluated, the w_1_ parameter capturing a tendency for social feedback to determine momentary feelings of self-worth did not correlate with global self-esteem (ρ(59)=-.13, p=.342). The only computational parameter that correlated with global self-esteem was the baseline self-worth parameter w_0_ (ρ(59)=.48, *p<*1×10^-4^) demonstrating that the w0 parameter captures baseline momentary self-worth during both evaluations of the self and evaluations of another person. The absence of differences between high and low self-esteem in terms of learning rate, expected approval rate and weight on social approval prediction errors, suggest that the behavioral differences found in the fMRI task reflect specific anomalies in how low self-esteem individuals learn from social feedback directed at the self rather than general impairments in (social) learning.

**Generosity toward raters in separate Dictator Games**

After the “other evaluation” task, participants played 12 independent economic games called Dictator games^6^. In each Dictator Game participants were endowed with £5 and were given the opportunity to anonymously transfer any amount of their endowment to another person and keep the remainder. They were instructed that one game would be randomly selected for payout. In Dictator games the recipient has no power over the distribution of money and passively receives any amount the distributor is willing to transfer. To reduce potential concerns about reputation, participants were told that money allocated to the other people would be anonymously added to their earnings. Participants played these Dictator games with 4 raters who gave approval feedback (1 from each group), 4 raters who gave disapproval feedback (1 from each group) and 4 raters for whom feedback was not displayed (1 from each group).

Participants donated on average 31% of their endowment to the raters showing they acted prosocial toward raters at their own financial cost (see Fig. S7). To test whether donation behavior was modulated by global self-esteem, social feedback and rater group, we performed a repeated-measures ANOVA on dictator game donations with self-esteem group (2 levels: high vs. low) as between-subjects factor and social feedback (3 levels: approval vs. disapproval vs. feedback not displayed) and rater group (4 levels: 87%, 67%, 33% and 13% approval) as within-subjects factors. This analysis revealed significant main effects of social feedback (*F*[2, 116]=81.48, *p<*1×10^-16^, η*_p_^2^*= .584) and rater group (*F*[3, 174]=5.10, *p=*.008, η*_p_^2^*= .081) and a significant interaction between social feedback and rater group (*F*[6, 348]=3.38, *p*=.009, η*_p_^2^*= .055). There was no significant main effect of self-esteem group (p=.115) or significant interactions with self-esteem (all *p*s>.084).

Follow-up pairwise comparisons showed that participants donated more money to raters who gave approval feedback (44% of the stake) than to raters for whom feedback was not displayed (30% of the stake, *p<*1×10^-9^) and raters who gave disapproval feedback (19% of the stake, *p<*1×10^-9^; see Fig. S7). Raters who gave approval feedback were treated similarly across the four rater groups (*F*(3, 177)=.763, *p*=.472, η*_p_^2^*= .013) as were raters who gave disapproval feedback (*F*(3, 177)=.474, *p*=.675, η*_p_^2^*= .008). However, raters for whom feedback was not displayed were treated differentially depending on group membership *F*(3, 177)=10.35, *p<* 1×10^-4^, η*_p_^2^*= .149). To be specific, participants transferred more money to raters from the 87% group (36% of the stake) than to the other 3 groups (all *p*s< .007). They also transferred more money to raters from the 67% group (31% of the stake) than the other 2 groups (20% and 22% respectively, both *p*s<.014). Participants donated similar amounts of money to raters from the 33% group and 13% group (p=.365). Together these results demonstrate that participants showed generosity toward raters typical of a generosity seen toward real humans^6^. Moreover, generosity was modulated by the type of feedback raters gave participants and raters’ general propensity to give approval feedback.

**Fig. S7*.* Generosity toward raters in independent Dictator Games in low (panel A) and high self-esteem (panel B) participants.** Participants completed 12 Dictator Games where they could donate a portion of a £5 endowment to recipients who had previously rated them in the social evaluation task. Donation behavior did not differ between low and self-esteem participants (*p*=.115). Participants donated more money to raters who gave approval feedback than to raters for whom feedback was not displayed (*p<*1×10^-9^) or raters who gave disapproval feedback (*p<*1×10^-9^). While group membership did not modulate donation behavior toward raters who gave approval (*p*=.472) or disapproval feedback (*p*=.675), group membership differentially impacted donation behavior toward raters for whom feedback was not displayed (*p<*1×10^-4^). When participants had no information about what raters thought of them, they donated most to raters from the 87% group (all *p*s<.007) followed by larger donations to raters from the 67% group compared to raters from the 33% group and 13% group (both *p*s<.014). One high self-esteem participant did not perform the Dictator Games due to technical issues. Data are represented as mean ± SEM.

**Neural signatures of social approval prediction errors**

To test whether participants with low and high self-esteem differed in neural representations of social approval prediction errors in ventral striatum/sgACC, we constructed a GLM that identified BOLD responses responding parametrically to social approval prediction errors upon receipt of feedback. BOLD responses in an independently defined region of interest (6-mm sphere surrounding peak coordinates of a cluster in ventral striatum/sgACC [5,20,-8] that responded to social approval prediction errors in an independent data set using the same paradigm^1^) correlated with social approval prediction errors (z=2.95, p=.003). We found no evidence for a difference in neural representation of social approval prediction errors between low and high self-esteem participants in this ROI (Mann-Whitney U test, z=-0.65, p=0.521; see Fig. S8) or at the whole-brain level at a significance level of FWE-corrected P<0.05.

**
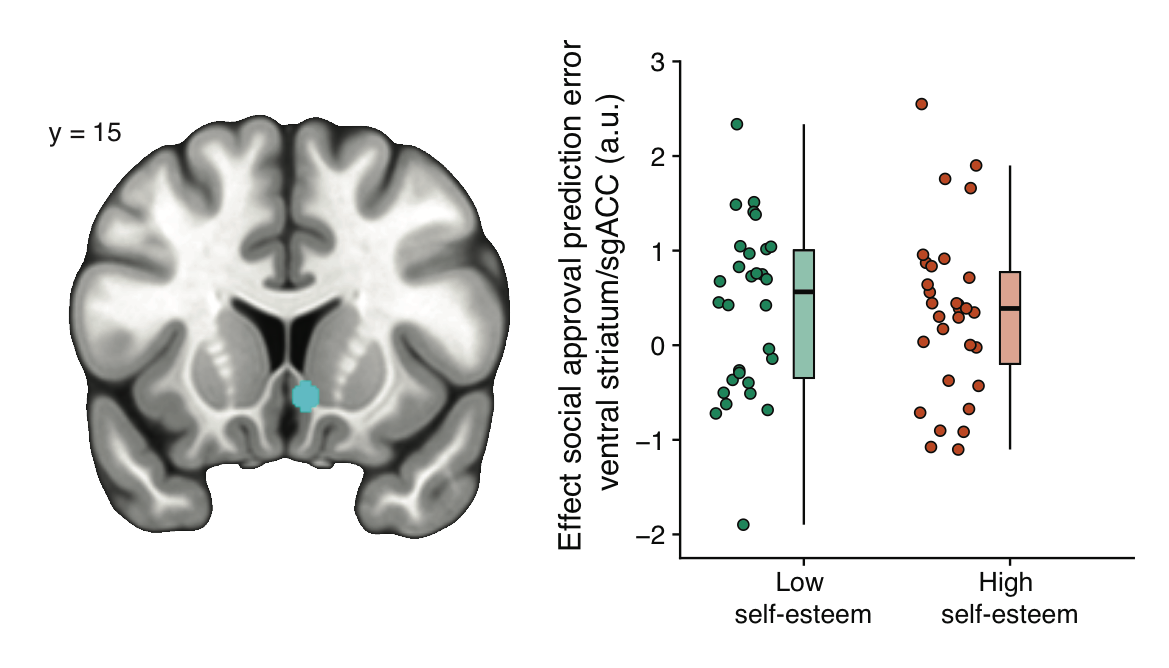
**

**Fig. S8. Neural representations of social approval prediction errors in high and low self-esteem participants.** Low self-esteem participants had similar neural responses to social approval prediction errors as high self-esteem participants (Mann-Whitney U test, z=-0.64, p=0.521) in an independently defined region of interest in ventral striatum/subgenual anterior cingulate found to respond to social approval prediction errors in a previous study^1^. Middle line of boxplots represents median and the lower and upper hinges of the boxes correspond to the first and third quartiles. The upper and lower whiskers extend from the hinge to the largest or smallest value respectively no further than 1.5 * interquartile range from the hinge.


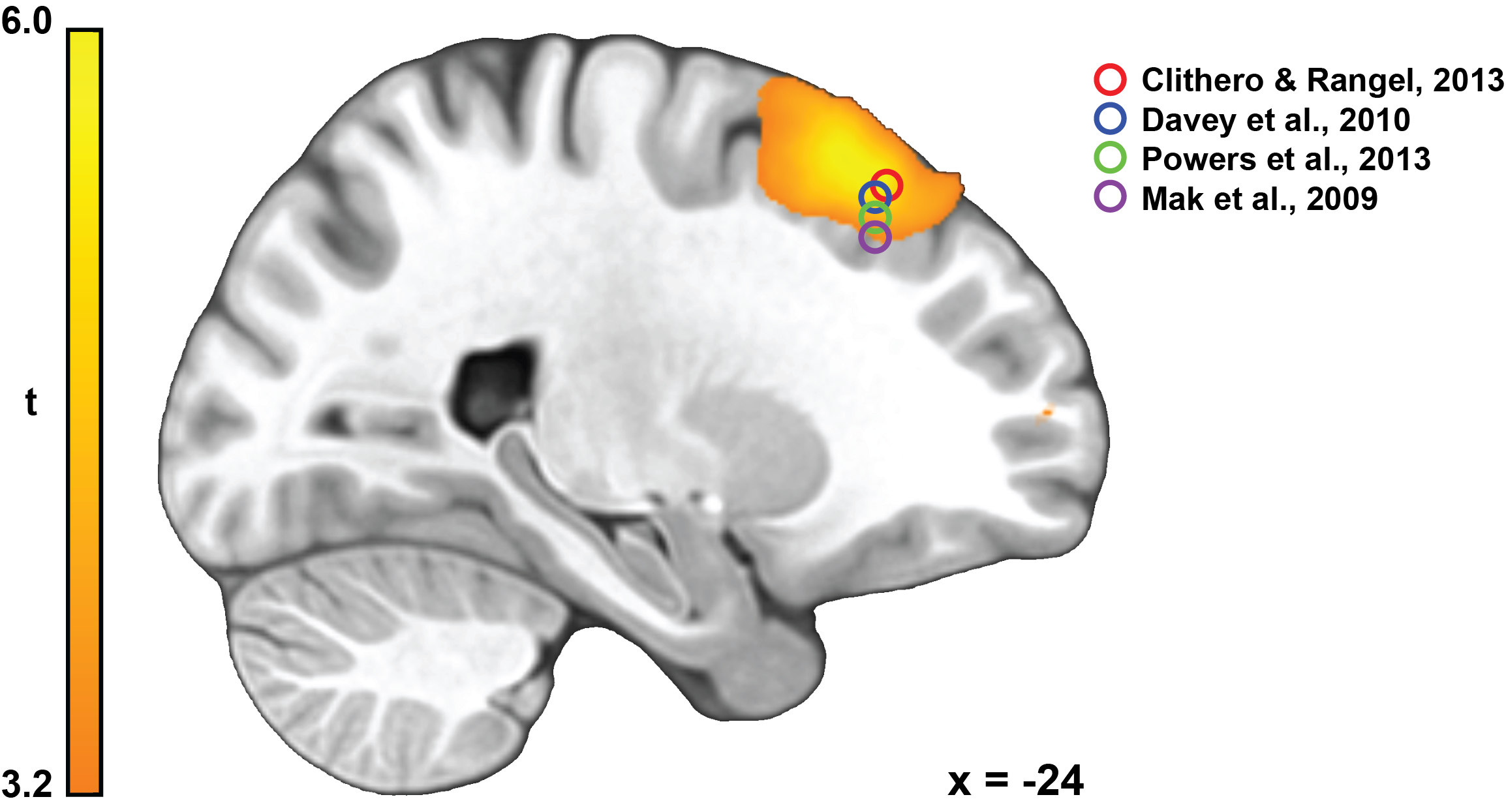


**Fig. S9**. **Function of dorsolateral superior frontal gyrus.** Peak coordinates from studies of social feedback processing (Davey et al., 2010; Powers et al., 2013), subjective value computations (Clithero & Rangel, 2010; meta-analysis of 81 fMRI studies) and emotion regulation (Mak et al., 2009) showing responses in Brodmann Area 8m in dorsolateral superior frontal gyrus. The warm color map shows significant activation in BA 8m in our study (whole brain regression analysis with trial-by-trial self-worth updates as parametric modulator; p=0.002, FWE cluster-corrected). Image is FWE cluster-corrected at p<0.05 (cluster-forming threshold of p<0.001) and overlaid on a high-resolution anatomical image.

Supplementary References

1. Will G-J, Rutledge RB, Moutoussis M, Dolan RJ. Neural and computational processes underlying dynamic changes in self-esteem. *eLife* 2017; **6**.

2. Allen M, Poggiali D, Whitaker K, Marshall TR, Kievit R. Raincloud plots: a multi-platform tool for robust data visualization. *PeerJ Preprints* 2018; **6:** e27137v27131.

3. Weiskopf N*, et al*. Quantitative multi-parameter mapping of R1, PD*, MT, and R2* at 3T: a multi-center validation. *Frontiers in neuroscience* 2013; **7:** 95.

4. Papp D, Callaghan MF, Meyer H, Buckley C, Weiskopf N. Correction of inter‐scan motion artifacts in quantitative R1 mapping by accounting for receive coil sensitivity effects. *Magnetic resonance in medicine* 2016; **76**(5)**:** 1478-1485.

5. Rescorla RA, Wagner AR. A theory of Pavlovian conditioning: Variations in the effectiveness of reinforcement and nonreinforcement. *Classical conditioning II: Current research and theory* 1972; **2:** 64-99.

6. Engel C. Dictator games: A meta study. *Experimental Economics* 2011; **14**(4)**:** 583-610.
